# Simultaneous Inference for High-Dimensional Approximate Factor Model

**DOI:** 10.3390/e22111258

**Published:** 2020-11-05

**Authors:** Yong Wang, Xiao Guo

**Affiliations:** 1 Department of Statistics and Finance, School of Management, University of Science and Technology of China, Hefei 230026, China; wy2608@mail.ustc.edu.cn; 2 International Institute of Finance, School of Management, University of Science and Technology of China, Hefei 230026, China

**Keywords:** high-dimensional factor model, multiple testing, multiplier bootstrap, simultaneous inference

## Abstract

This paper studies simultaneous inference for factor loadings in the approximate factor model. We propose a test statistic based on the maximum discrepancy measure. Taking advantage of the fact that the test statistic can be approximated by the sum of the independent random variables, we develop a multiplier bootstrap procedure to calculate the critical value, and demonstrate the asymptotic size and power of the test. Finally, we apply our result to multiple testing problems by controlling the family-wise error rate (FWER). The conclusions are confirmed by simulations and real data analysis.

## 1. Introduction

The high-dimensional factor model is becoming more and more important in different scientific areas including finance and macroeconomics. For example, the data in the World Bank contain two-hundred countries over forty years and the number of stocks can be in the thousands which is larger than or of the same order of the sample size for portfolio allocation. Due to its broad applications, much efforts have been devoted to analyzing factor model in different aspects. Examples include estimation of factors and loadings for latent factor model [1,2], covariance matrix estimation [3,4,5,6], and simultaneous inference for factor loadings of dynamic factor model [7,8], among others.

This work focuses on the simultaneous inference for the loading matrix with observed factors, which is an important issue in the analysis of approximate factor models. For example, in the study of gene expression genomics, it is commonly assumed that each gene is associated with only a few factors. For example, the authors of [9] showed that several oncogenes are related to Rb/E2F pathway rather than any other pathways. The authors of [10] also considered sparse loading matrix for gene expression data. Therefore, it is necessary to test the sparsity of the factor loadings. In the literature, some inference procedures have been proposed for latent factor models. For example, in low-dimensional setting, the authors of [11] considered testing the homogeneity assumption, i.e., the loadings associated to a factor are identical for all variables. The same testing problem has been considered by the authors of [12] in high-dimensional situation. As for observed factors, to the best of our knowledge, very limited work has been conducted.

Inference for the factor loadings with observed factors is not trivial. The approaches for latent factors cannot be directly applied to observed factors. The major difficulty is due to the high dimensionality, which poses significant challenges in deriving the asymptotic null limiting distribution of the test statistic. We propose a test statistic based on the maximum discrepancy measure. The distribution of this statistic is attractive in high-dimensional statistical inference such as model selection, simultaneous inference, and multiple testing. Examples include the works in [13,14,15,16,17], among others.

We use the multiplier bootstrap procedure to obtain the critical value of our test statistic. Based on the fact that the test statistic can be approximated by the sum of the independent random variables, we show that the proposed multiplier bootstrap method consistently approximates the null limiting distribution of the test statistic, and thus the testing procedure achieves the prespecified significance level asymptotically. There are some related works applying multiplier bootstrap method to high-dimensional inference; see in [16,18,19], among others. However, their procedures require sparsity assumption on the parameters and cannot be directly applied to factor model. Compared with the works with latent factors, we do not require homogeneity constraints or sparsity on the model and our procedure is adaptive to high-dimensional regime.

Another application of our procedure is the multiple testing problem. Combining the multiplier bootstrap method with step-down procedure proposed by [17], we show that our procedure has a strong control of the family-wise error rate (FWER). Our method is asymptotically non-conservative as compared to the Bonferroni–Holm procedure since the correlation among the test statistics has been taken into account. We also want to point out that any procedure controlling the FWER will also control the false discovery rate [20] when there exist some true discoveries.

The rest of the paper is organized as follows. In Section 2.1, we develop the multiplier bootstrap procedure for simultaneous test of parameters for a single factor and demonstrate its asymptotic level and power. In Section 2.2, we give the result of simultaneous test of parameters for multiple factors. Section 3 discusses the multiple testing problem by combining the multiplier bootstrap procedure with the step-down method proposed by [17]. Section 4 investigates the numerical performance of the proposed test by simulations. We also conduct real data analysis on portfolio risk of S&P stocks via Fama–French model in Section 5. The proofs of the main results are given in Appendix A.

Finally, we introduce some notation. For set *S*, let |S| denote the cardinality of *S*. Let $0_{p}\in R^{p}$ be the vector of zeros. For p×p matrix $A={\left(a_{ij}\right)}_{i,j=1}^{p}$, denote by $\lambda_{\min}\left(A\right)$ and $\lambda_{\max}\left(A\right)$ the minimum and maximum eigenvalues of A, respectively. The matrix element-wise maximum norm and $L_{2}$ norm are defined as ${∥A∥}_{\infty }=\max_{1\leq i,j\leq p}\left|a_{ij}\right|$ and $∥A∥=\lambda_{\max}^{1/2}\left(A^{\prime }A\right)$, respectively. For $a={\left(a_{1},\cdots ,a_{p}\right)}^{\prime }\in R^{p}$ and q>0, denote by ${∥a∥}_{q}=(\sum_{i=1}^{p}|a_{i}{{|}^{q})}^{1/q}$ and ${∥a∥}_{\infty }=\max_{1\leq j\leq p}\left|a_{j}\right|$. Let $v_{i}\in R^{K}$ be the *i*th column of the K×K identity matrix. We write $a_{t}≲b_{t}$ if $a_{t}$ is smaller than or equal to $b_{t}$ up to a universal positive constant. For a,b∈R, we write a∨b=max{a,b}. For two sets, *A* and *B*, A⊖B denotes their symmetric difference, that is, A⊖B=(A∖B)∪(B∖A).

## 2. Methodology

### 2.1. Simultaneous Test for a Single Factor

We consider the factor model defined as follows,

(1) $$y_{it}=b_{i}^{\prime }f_{t}+u_{it},\;i=1,\cdots ,p\;\mathrm{and}\;t=1,\cdots ,T,$$

where $y_{it}$ is the observed response for the *i*th variable at time *t*, $b_{i}\in R^{K}$ is the unknown vector of factor loadings, $f_{t}\in R^{K}$ is the observed vector of common factors, and $u_{it}$ is the latent error. Here, *K* is a fixed integer denoting the number of factors, *p* is the number of variables, and *T* denotes the sample size. Model (1) is commonly used in finance and macroeconomics, see, e.g., in [3,4,21], among others.

Denote by $B={\left(b_{1},\cdots ,b_{p}\right)}^{\prime }$, $y_{t}={\left(y_{1t},\cdots ,y_{pt}\right)}^{\prime }$ and $u_{t}={\left(u_{1t},\cdots ,u_{pt}\right)}^{\prime }$, then model (1) can be re-expressed as

(2) $$y_{t}=Bf_{t}+u_{t}.$$

We first focus on testing the coefficients $b_{ik}=b_{i}^{\prime }v_{k}$ corresponding to a single factor, i.e., the *k*th factor. Specifically, we consider the following simultaneous testing problem that given k=1,…,K,

(3) $$H_{0,G}:b_{ik}=b_{ik}^{\mathrm{null}}\;\;\mathrm{for}\;\mathrm{all}\;i\in G\;\mathrm{versus}\;H_{1,G}:b_{ik}\neq b_{ik}^{\mathrm{null}}\;\mathrm{for}\;\mathrm{some}\;i\in G,$$

where *G* is a subset of {1,⋯,p} and $b_{ik}^{\mathrm{null}}$ are prespecified values. For example, if $b_{ik}^{\mathrm{null}}$ are 0, then the hypotheses are able to test whether the variables with indices in *G* are significantly associated with the *k*th factor. Throughout the paper, |G| is allowed to grow as fast as *p*, which may have exponential growth with *T* as in Assumption 3.

The ordinary least squares (OLS) estimator $\hat{B}=Y^{\prime }F{\left(F^{\prime }F\right)}^{-1}$ is applied to estimate B, where $Y={\left(y_{1},\ldots ,y_{T}\right)}^{\prime }$ and $F={\left(f_{1},\ldots ,f_{T}\right)}^{\prime }$. Therefore,

(4) $$\hat{B}-B=\left(\sum_{t=1}^{T}u_{t}f_{t}^{\prime }\right){\left(\sum_{t=1}^{T}f_{t}f_{t}^{\prime }\right)}^{-1}.$$

We propose the following test statistic for $H_{0,G}$,

$$M_{T,k}=\max_{i\in G}\sqrt{T}\left|{\hat{b}}_{ik}-b_{ik}^{\mathrm{null}}\right|,$$

where ${\left({\hat{b}}_{ik}\right)}_{i\leq p,k\leq K}=\hat{B}$. For each i∈G, the asymptotic normality of ${\hat{b}}_{ik}$ is straightforward due to the central limit theorem. However, when |G| diverges with *p*, it is very challenging to demonstrate the existence of the limiting distribution of $M_{T,k}$. In order to approximate the asymptotic distribution of $M_{T,k}$, we will use the multiplier bootstrap method. From (4), we know

(5) $$\sqrt{T}\left({\hat{b}}_{ik}-b_{ik}\right)=\frac{1}{\sqrt{T}}\sum_{t=1}^{T}u_{it}f_{t}^{\prime }{\hat{\Omega }}_{f}v_{k}=\frac{1}{\sqrt{T}}\sum_{t=1}^{T}{\hat{\xi }}_{it},$$

where ${\hat{\xi }}_{it}=u_{it}f_{t}^{\prime }{\hat{\Omega }}_{f}v_{k}$ and ${\hat{\Omega }}_{f}={\left(\sum_{t=1}^{T}f_{t}f_{t}^{\prime }/T\right)}^{-1}$.

In order to apply the multiplier bootstrap procedure, we need to approximate $\sum_{t=1}^{T}{\hat{\xi }}_{it}/\sqrt{T}$ by sum of independent random variables. As ${\hat{\Omega }}_{f}$ is consistent for $\Omega_{f}={\left\{E\left(f_{t}f_{t}^{\prime }\right)\right\}}^{-1}$, we can replace the former with the latter in ${\hat{\xi }}_{it}$, and define $\xi_{it}=u_{it}f_{t}^{\prime }\Omega_{f}v_{k}$. Then, for each i∈G, ${\left\{\xi_{it}\right\}}_{t\geq 1}$ are i.i.d. and $\sum_{t=1}^{T}\xi_{it}/\sqrt{T}$ well approximates $\sum_{t=1}^{T}{\hat{\xi }}_{it}/\sqrt{T}$.

We then apply the multiplier bootstrap procedure to approximate the distribution of $\max_{i\in G}\left|\sum_{t=1}^{T}\xi_{it}\right|/\sqrt{T}$. Denote by $\Sigma_{u}={\left(\sigma_{ij}\right)}_{p\times p}$ the covariance matrix of $u_{t}$, and hence $\mathrm{cov}\left(\xi_{it},\xi_{jt}\right)=\Omega_{f}\left(k,k\right)\sigma_{ij}$, where $\Omega_{f}\left(k,k\right)=v_{k}^{\prime }\Omega_{f}v_{k}$. We know that ${\hat{\Omega }}_{f}\left(k,k\right)=v_{k}^{\prime }{\hat{\Omega }}_{f}v_{k}$ is $\sqrt{T}$-consistent for $\Omega_{f}\left(k,k\right)$. To estimate $\sigma_{ij}$, we first calculate the residuals

$${\hat{u}}_{it}=y_{it}-{\hat{b}}_{i}^{\prime }f_{t}.$$

Denote by ${\hat{u}}_{t}={\left({\hat{u}}_{1t},\cdots ,{\hat{u}}_{pt}\right)}^{\prime }$, then the error covariance matrix is estimated by

$${\hat{\Sigma }}_{u}=\frac{1}{T}\sum_{t=1}^{T}{\hat{u}}_{t}{\hat{u}}_{t}^{\prime }={\left({\hat{\sigma }}_{ij}\right)}_{i\leq p,j\leq p}.$$

Let ${\left\{e_{t}\right\}}_{t=1}^{T}$, a sequence of i.i.d. N(0,1) independent of ${\left\{y_{t},f_{t}\right\}}_{t=1}^{T}$, be the multiplier random variables. Then the multiplier bootstrap statistic is defined as

$$W_{T,k}=\max_{i\in G}T^{-1/2}\sqrt{{\hat{\Omega }}_{f}\left(k,k\right)}|\sum_{t=1}^{T}{\hat{u}}_{it}e_{t}|.$$

Conditioning on ${\left\{y_{t},f_{t}\right\}}_{t=1}^{T}$, the covariance of $T^{-1/2}\sum_{t=1}^{T}\sqrt{{\hat{\Omega }}_{f}\left(k,k\right)}{\hat{u}}_{it}e_{t}$ and $T^{-1/2}\sum_{t=1}^{T}\sqrt{{\hat{\Omega }}_{f}\left(k,k\right)}{\hat{u}}_{jt}e_{t}$ is ${\hat{\Omega }}_{f}\left(k,k\right){\hat{\sigma }}_{ij}$, which can sufficiently approximate the covariance between $\xi_{it}$ and $\xi_{jt}$. Then, the bootstrap critical value can be obtained via

$$c_{W_{T,k}}\left(\alpha \right)=\inf\left\{t\in R:P\left(W_{T,k}\leq t|\left(Y,F\right)\right)\geq 1-\alpha \right\}.$$

$c_{W_{T,k}}\left(\alpha \right)$ is calculated by generating ${\left\{e_{t}\right\}}_{t=1}^{T}$ repeatedly. In our simulations and real data, we conduct bootstrap 500 times. We now present some technical assumptions.

**Assumption** **1.**

(i) *${\left\{f_{t},u_{t}\right\}}_{t\geq 1}$ are i.i.d. with $E\left(u_{t}\right)=0_{p}$ and $\Sigma_{u}=\mathrm{cov}\left(u_{t}\right)$.*
(ii) *There exist constants $c_{1},c_{2}$ such that $0<c_{1}<\lambda_{\min}\left(\Sigma_{u}\right)<\lambda_{\max}\left(\Sigma_{u}\right)<c_{2}<\infty$.*
(iii) *${\left\{u_{t}\right\}}_{t\geq 1}$ and ${\left\{f_{t}\right\}}_{t\geq 1}$ are independent.*

**Assumption** **2.** *There exist positive constants $r_{1},r_{2},b_{1},b_{2}$, such that for any s>0, t≤T, i≤p and j≤K,*

$$P(|u_{it}\left|>s\right)\leq \exp\left\{-{\left(s/b_{1}\right)}^{r_{1}}\right\},\;P(|f_{jt}\left|>s\right)\leq \exp\left\{-{\left(s/b_{2}\right)}^{r_{2}}\right\}.$$

The “i.i.d.” condition in Assumption 1 is commonly considered in the literature for high-dimensional inference, see, e.g., in [16]. Assumption 1 (ii) allows the bounded eigenvalue of the error covariance matrix. As noted in [22], such assumption is satisfied by two situations: (1) $\mathrm{cov}(U_{1},\cdots ,U_{p})$, where $\left\{U_{i},\;i\geq 1\right\}$ is a stationary ergodic process with spectral density *f*, $0<c_{1}<f<c_{2}$ and (2) $\mathrm{cov}(X_{1},\cdots ,X_{p})$ where $X_{i}=U_{i}+V_{i},\;i=1,\cdots ,p$, $\left\{U_{i}\right\}$ is a stationary process as above and $\left\{V_{i}\right\}$ is a noise process independent of $\left\{U_{i}\right\}$. In Example 1 in [22], they demonstrated that ARMA(r,q) process satisfies Assumption 1 (ii). Furthermore, this assumption is commonly considered in the literature, see, e.g., in [4,15].

Assumption 2 allows the application of the large deviation theory to $\left(1/T\right)\sum_{t=1}^{T}u_{it}u_{jt}-\sigma_{ij}$ and $\left(1/T\right)\sum_{t=1}^{T}u_{it}f_{jt}$. In this paper, we assume that $f_{t}$ and $u_{t}$ have exponential-type tails. Let $\gamma_{1}^{-1}=3r_{1}^{-1}$ and $\gamma_{2}^{-1}=1.5r_{1}^{-1}+1.5r_{2}^{-1}$.

**Assumption** **3.** *Suppose $\gamma_{1}<1$, $\gamma_{2}<1$ and there exists a constant $c_{1}>0$, such that ${\left(\log p\right)}^{\gamma }=o\left(T\right)$, where $\gamma =\max\{2/\gamma_{1}-1,2/\gamma_{2}-1,7+c_{1}\}$.*

**Assumption** **4.** *There exists a constant C>0 such that $\lambda_{\max}\left(\Omega_{f}\right)<C$.*

Assumption 3 is needed in Bernstein-type inequality [23] and commonly assumed in the literature for Gaussian approximation theory. Assumption 4 is also reasonable by bounding the eigenvalues of $\Omega_{f}$.

**Theorem** **1.** *Under Assumptions 1–4, we have*

$$\underset{\alpha \in (0,1)}{\sup}|P\left(\max_{i\in G}\sqrt{T}\left|{\hat{b}}_{ik}-b_{ik}\right|>c_{W_{T,k}}\left(\alpha \right)\right)-\alpha |=o\left(1\right).$$

Theorem 1 demonstrates that the multiplier bootstrap critical value $c_{W_{T,k}}\left(\alpha \right)$ well approximates the quantile of the test statistic. It is worth mentioning that our method does not require any sparsity assumption on either $\Sigma_{u}$ or B.

The proof of Theorem 1 depends on the two results: (1) $\max_{i\in G}\sum_{t=1}^{T}\xi_{it}/\sqrt{T}$ is sufficiently close to $\max_{i\in G}\sum_{t=1}^{T}{\hat{\xi }}_{it}/\sqrt{T}$ and (2) the covariances of $\xi_{it}$ and $\xi_{jt}$ are well approximated by the bootstrap version. The first result is demonstrated in Lemma A7 that there exist $\zeta_{1}>0$ and $\zeta_{2}>0$ such that

$$P\left(|\max_{i\in G}\sum_{t=1}^{T}{\hat{\xi }}_{it}/\sqrt{T}-\max_{i\in G}\sum_{t=1}^{T}\xi_{it}/\sqrt{T}|>\zeta_{1}\right)<\zeta_{2},$$

where $\zeta_{1}\sqrt{1\vee \log(p/\zeta_{1})}=o\left(1\right)$ and $\zeta_{2}=o\left(1\right)$. The second result is shown in Lemma A6 that

$$\Delta =\max_{1\leq i,j\leq p}\left|{\hat{\Omega }}_{f}\left(k,k\right){\hat{\sigma }}_{ij}-\Omega_{f}\left(k,k\right)\sigma_{ij}\right|=o_{P}\left({\left(\log p\right)}^{-2}\right),$$

i.e., the maximum discrepancy between the empirical and population covariance matrices converges to zero.

Based on Theorem 1, for a given significance level 0<α<1, we define the test $\Phi_{\alpha }$ by

(6) $$\Phi_{\alpha }=I\left(M_{T,k}>c_{W_{T,k}}\left(\alpha \right)\right).$$

The hypothesis $H_{0,G}$ is rejected whenever $\Phi_{\alpha }=1$.

Bootstrap is a commonly used resampling method and full theories about it can be found in [24]. There are many versions of bootstrap, for example, wild bootstrap, empirical bootstrap, and multiplier bootstrap, among others. As discussed in [25], other exchangeable bootstrap methods are asymptotically equivalent to the multiplier bootstrap. As our test statistic can be approximated by the maximum of sum of independent random vectors, we adopt the multiplier bootstrap method in [25] based on Gaussian approximation.

Alternatively, we propose the studentized statistic $M_{T,k}^{*}:=\max_{i\in G}\sqrt{T}\left|{\hat{b}}_{ik}-b_{ik}^{\mathrm{null}}\right|/\sqrt{{\hat{\omega }}_{ii}}$ for $H_{0,G}$, where ${\hat{\omega }}_{ii}={\hat{\Omega }}_{f}\left(k,k\right){\hat{\sigma }}_{ii}$. Similarly to Section 2.1, we define the multiplier bootstrap statistic as

$$W_{T,k}^{*}=\max_{i\in G}T^{-1/2}|\sum_{t=1}^{T}{\hat{u}}_{it}e_{t}|\sqrt{{\hat{\Omega }}_{f}\left(k,k\right)/{\hat{\omega }}_{ii}}=\max_{i\in G}T^{-1/2}{\hat{\sigma }}_{ii}^{-1/2}|\sum_{t=1}^{T}{\hat{u}}_{it}e_{t}|,$$

where ${\left\{e_{t}\right\}}_{t=1}^{T}\overset{i.i.d.}{∼}N\left(0,1\right)$ are independent of ${\left\{y_{t},f_{t}\right\}}_{t=1}^{T}$. Then, the bootstrap critical value can be obtained via

$$c_{W_{T,k}}^{*}\left(\alpha \right)=\inf\left\{t\in R:P\left(W_{T,k}^{*}\leq t|\left(Y,F\right)\right)\geq 1-\alpha \right\}.$$

Theorem 2 below justifies the validity of the bootstrap procedure for the studentized statistic.

**Theorem** **2.** *Under the assumptions in Theorem 1, we have*

$$\underset{\alpha \in (0,1)}{\sup}|P\left(\max_{i\in G}\sqrt{T}\left|{\hat{b}}_{ik}-b_{ik}\right|/\sqrt{{\hat{\omega }}_{ii}}>c_{W_{T,k}}^{*}\left(\alpha \right)\right)-\alpha |=o\left(1\right).$$

Based on this result, for a given significance level 0<α<1, we define the test $\Phi_{\alpha }^{*}$ by

$$\Phi_{\alpha }^{*}=I\left(M_{T,k}^{*}>c_{W_{T,k}}^{*}\left(\alpha \right)\right).$$

The hypothesis $H_{0,G}$ is rejected whenever $\Phi_{\alpha }^{*}=1$.

For the studentized statistic, we can derive its asymptotic distribution. By Lemma 6 of the work in [15], for any x∈R and as p→∞, we have

$$P\left(\max_{1\leq i\leq p}T{\left|{\hat{b}}_{ik}-b_{ik}\right|}^{2}/{\hat{\omega }}_{ii}-2\log\left(p\right)+\log\log\left(p\right)\leq x\right)\to \exp\left\{-\frac{1}{\sqrt{\pi }}\exp\left(-\frac{x}{2}\right)\right\}.$$

However, the above alternative testing procedure may not perform well in practice, because it requires diverging *p*, and the convergence rate is typically slow.

In contrast to the extreme value approach, our testing procedure explicitly accounts for the effect of |G| in the sense that the bootstrap critical value $c_{W_{T,k}}^{*}\left(\alpha \right)$ depends on *G*. Therefore, our approach is more robust to the change in |G|.

Next, we turn to the (asymptotic) power analysis of the above procedure. Denote by $B_{k}$ the *k*th column of B. We focus on the case where |G|→∞ as T→∞ below. Define the separation set

(7) $$U_{G}\left(c\right)=\{{\left(b_{1k},\cdots ,b_{pk}\right)}^{T}:\max_{i\in G}|b_{ik}-b_{ik}^{\mathrm{null}}\left|/\sqrt{\omega_{ii}}>c\sqrt{\log(|G|)/T}\right\},$$

where $\omega_{ij}=\Omega_{f}\left(k,k\right)\sigma_{ij}$. Let $\Theta ={\left(\theta_{ij}\right)}_{i,j=1}^{p}$ with $\theta_{ij}=\omega_{ij}/\sqrt{\omega_{ii}\omega_{jj}}=\sigma_{ij}/\sqrt{\sigma_{ii}\sigma_{jj}}$, which is the correlation matrix of $u_{t}$.

**Assumption** **5.** *Suppose $\max_{1\leq i\neq j\leq p}\left|\theta_{ij}\right|\leq c_{0}<1$ for some constant $c_{0}$.*

**Theorem** **3.** *Under Assumptions 1–5, for any $\varepsilon_{0}>0$, we have*

$$\underset{B_{k}\in U_{G}\left(\sqrt{2}+\varepsilon_{0}\right)}{\inf}P\left(\max_{i\in G}\sqrt{T}\left|{\hat{b}}_{ik}-b_{ik}^{\mathrm{null}}\right|/\sqrt{{\hat{\omega }}_{ii}}>c_{W_{T,k}}^{*}\left(\alpha \right)\right)\to 1.$$

As long as one entry of $b_{ik}-b_{ik}^{\mathrm{null}}$ has a magnitude larger than $\left(\sqrt{2}+\varepsilon_{0}\right)\sqrt{\log|G|/T}$, our bootstrap-assisted test can reject the null correctly. Therefore, with B being sparse, our procedure performs well in detecting non-sparse alternatives. According to Section 3.2 of [26], the separation rate $\left(\sqrt{2}+\varepsilon_{0}\right)\sqrt{\log(|G|)/T}$ is minimax optimal under suitable assumptions.

### 2.2. Simultaneous Test for Multiple Factors

In this section, we test the elements of the loading matrix corresponding to different factors. The testing problem can be stated as follows,

$$H_{0,G^{*}}:b_{ik}=b_{ik}^{\mathrm{null}}\;\;\mathrm{for}\;\mathrm{all}\;\left(i,k\right)\in G^{*}\;\mathrm{versus}\;H_{1,G^{*}}:b_{ik}\neq b_{ik}^{\mathrm{null}}\;\mathrm{for}\;\mathrm{some}\;\left(i,k\right)\in G^{*},$$

where $G^{*}$ is a subset of M≡{(i,j):i=1,⋯pandj=1,⋯,K}. Define

$$\begin{array}{ccc} \omega_{\left(i,k),(j,\ell \right)}^{*} & = & \mathrm{cov}\left(u_{it}f_{t}^{\prime }\Omega_{f}v_{k},u_{jt}f_{t}^{\prime }\Omega_{f}v_{\ell }\right)=\sigma_{ij}v_{k}^{\prime }\Omega_{f}v_{\ell },\\ {\hat{\omega }}_{\left(i,k),(j,\ell \right)}^{*} & = & {\left({\hat{\Omega }}_{f}v_{k}\right)}^{\prime }\left(\frac{1}{T}\sum_{t=1}^{T}{\hat{u}}_{it}{\hat{u}}_{jt}f_{t}f_{t}^{\prime }\right)\left({\hat{\Omega }}_{f}v_{\ell }\right). \end{array}$$

We propose the studentized test statistic

$$M_{T,G^{*}}=\max_{\left(i,k\right)\in G^{*}}\sqrt{T}\left|{\hat{b}}_{ik}-b_{ik}\right|/\sqrt{{\hat{\omega }}_{\left(i,k),(i,k\right)}^{*}}.$$

From the linear expansion in (5), the multiplier bootstrap statistic is defined as

$$W_{T,G^{*}}=\max_{\left(i,k\right)\in G^{*}}T^{-1/2}|\sum_{t=1}^{T}{\hat{u}}_{it}f_{t}^{\prime }{\hat{\Omega }}_{f}v_{k}e_{t}|/\sqrt{{\hat{\omega }}_{\left(i,k),(i,k\right)}^{*}},$$

where ${\left\{e_{t}\right\}}_{t=1}^{T}\overset{i.i.d.}{∼}N\left(0,1\right)$ are independent of ${\left\{y_{t},f_{t}\right\}}_{t=1}^{T}$. Then, the bootstrap critical value can be obtained via

$$c_{W_{T,G^{*}}}\left(\alpha \right)=\inf\left\{t\in R:P\left(W_{T,G^{*}}\leq t|\left(Y,F\right)\right)\geq 1-\alpha \right\}.$$

Let $\gamma_{3}^{-1}=4r_{1}^{-1}+4r_{2}^{-1}$, $r_{3}^{-1}=3r_{1}^{-1}+9r_{2}^{-1}$ and $r=\max\{2/\gamma_{3}-1,2/r_{3}-1,c_{1}+7\}$ for a constant $c_{1}>0$.

**Theorem** **4.** *Suppose ${\left(\log p\right)}^{r}=o\left(T\right)$, under Assumptions 1,2 and 4, we have*

$$\underset{\alpha \in (0,1)}{\sup}|P\left(\max_{\left(i,k\right)\in G^{*}}\sqrt{T}\left|{\hat{b}}_{ik}-b_{ik}\right|/\sqrt{{\hat{\omega }}_{\left(i,k),(i,k\right)}^{*}}>c_{W_{T,G^{*}}}\left(\alpha \right)\right)-\alpha |=o\left(1\right).$$

Based on Theorem 4, for a given significance level 0<α<1, we define the test $\Phi_{\alpha }\left(G^{*}\right)$ by

$$\Phi_{\alpha }\left(G^{*}\right)=I\left(M_{T,G^{*}}>c_{W_{T,G^{*}}}\left(\alpha \right)\right).$$

The hypothesis $H_{0,G^{*}}$ is rejected whenever $\Phi_{\alpha }\left(G^{*}\right)=1$.

Now we turn to the power analysis of the test $\Phi_{\alpha }\left(G^{*}\right)$. Similar to Section 2.1, we focus on the case where $|G^{*}|\to \infty$ as T→∞ and define the separation set

$$V_{G^{*}}\left(c\right)=\{{\left(b_{ik}\right)}_{i\leq p,k\leq K}:\max_{\left(i,k\right)\in G^{*}}|b_{ik}-b_{ik}^{\mathrm{null}}\left|/\sqrt{\omega_{\left(i,k),(i,k\right)}^{*}}>c\sqrt{\log(|G^{*}|)/T}\right\},$$

Let $\theta_{\left(i,k),(j,\ell \right)}^{*}=\omega_{\left(i,k),(j,\ell \right)}^{*}/\sqrt{\omega_{\left(i,k),(i,k\right)}^{*}\omega_{\left(j,\ell ),(j,\ell \right)}^{*}}$. We consider the following condition.

**Assumption** **6.** Suppose $\max_{\left(i,k),(j,\ell \right)}\left|\theta_{\left(i,k),(j,\ell \right)}^{*}\right|\leq c_{0}^{*}<1$ for some constant $c_{0}^{*}$.

The asymptotic power of the testing procedure is given as follows.

**Theorem** **5.** *Under the assumptions in Theorem 4 and Assumption 6 , for any $\varepsilon_{0}>0$, we have*

$$\underset{B\in V_{G^{*}}\left(\sqrt{2}+\varepsilon_{0}\right)}{\inf}P\left(\max_{\left(i,k\right)\in G^{*}}\sqrt{T}\left|{\hat{b}}_{ik}-b_{ik}^{\mathrm{null}}\right|/\sqrt{{\hat{\omega }}_{\left(i,k),(i,k\right)}^{*}}>c_{W_{T,G^{*}}}\left(\alpha \right)\right)\to 1.$$

## 3. Multiple Testing with Strong FWER Control

In this section, we study the following multiple testing problem,

$$H_{0,i}:b_{ij}\leq b_{ij}^{\mathrm{null}}\;\;\mathrm{versus}\;\;H_{1,i}:b_{ij}>b_{ij}^{\mathrm{null}}\;\;\mathrm{for}\;\mathrm{all}\;i\in G.$$

For simplicity, we set G={1,2,⋯,p} and let *j* be fixed. We combine the bootstrap-assisted procedure with the step-down method proposed by [17]. Our method can be seen as a special case in Section 5 of [25]. Note that this framework can cover the case of testing equalities $\left(H_{0,j}:b_{ij}=b_{ij}^{\mathrm{null}}\right)$ because equalities can be rewritten as pairs of inequalities.

We briefly illustrate the control of the FWER. Full details and theory can be found in [25]. Let Ω be the space for all data generating processes, and ω be the true process. Each null hypothesis $H_{0,i}$ is equivalent to $\omega \in \Omega_{i}$ for some $\Omega_{i}\subset \Omega$. For any η⊂G, denote by $\Omega^{\eta }=\left(\cap_{i\in \eta }\Omega_{i}\right)\cap \left(\cap_{i\notin \eta }\Omega_{i}^{c}\right)$ with $\Omega_{i}^{c}=\Omega \setminus \Omega_{i}$. The strong control of the FWER means that

(8) $$\underset{\eta \subset G}{\sup}\underset{\omega \in \Omega^{\eta }}{\sup}P_{\omega }\left(\;\mathrm{reject}\;\mathrm{at}\;\mathrm{least}\;\mathrm{one}\;\mathrm{hypothesis}\;H_{0,i},\;i\in \omega \right)\leq \alpha +o\left(1\right),$$

where $P_{\omega }$ denotes the probability distribution under the data-generating process ω.

For i=1,⋯,p, denote $t_{ij}=\sqrt{T}\left({\hat{b}}_{ij}-b_{ij}^{\mathrm{null}}\right)$. For a subset η⊂G, let $c_{\eta }\left(\alpha \right)$ be the bootstrapped estimate for the (1−α)-quantile of $\max_{i\in \eta }t_{ij}$. The step-down procedure in [17] is described as follows. Define η(1)=G at the first step and reject all $H_{0,i}$ satisfying $t_{ij}>c_{\eta (1)}\left(\alpha \right)$. If no $H_{0,i}$ is rejected, then stop the procedure. If some $H_{0,i}$ are rejected, let η(2) be the set of indices for those hypotheses not being rejected at the first step. On step ℓ≥2, let η(ℓ)⊂G be the subset of hypotheses that were not rejected at step ℓ−1. Reject all hypotheses $H_{0,i}$ for i∈η(ℓ) satisfying that $t_{ij}>c_{\eta (\ell )}\left(\alpha \right)$. If no hypothesis is rejected, then stop the procedure. Proceed in this way until the algorithm stops.

Romano and Wolf [17] proved the following result:

(9) $$c_{\eta }\left(\alpha \right)\leq c_{\eta^{\prime }}\left(\alpha \right),\;\;\mathrm{for}\;\eta \subset \eta^{\prime }$$

(10) $$\underset{\eta \subset G}{\sup}\underset{\omega \in \Omega^{\eta }}{\sup}P_{\omega }\left(\max_{i\in \eta }t_{ij}>c_{\eta }\left(\alpha \right)\right)\leq \alpha +o\left(1\right).$$

Therefore, we can show that the step-down method together with the multiplier bootstrap provide strong control of the FWER by verifying (9) and (10). The theoretical results are given in the proposition below. The proofs are similar to those of Theorem 1, which are omitted here.

**Proposition** **1.** *Under the assumptions in Theorem 1, the step-down procedure with the bootstrap critical value $c_{\eta }\left(\alpha \right)$ satisfies (8).*

Our multiple testing method has the following two important features: (i) It can be applied to models with an increasing dimension; (ii) It takes into account the correlation amongst statistics and hence is asymptotically non-conservative.

In the simulation, we also consider Benjamini–Hochberg procedure [20] to control the false discovery rate (FDR), which is summarized as follows. For each of $H_{0,1},\cdots ,H_{0,p}$, we calculate the *p*-values $P_{1},\cdots ,P_{p}$ based on the studentized test statistic. Let $P_{\left(1\right)}\leq \cdots \leq P_{\left(p\right)}$ be the ordered *p*-values, and denote by $H_{0,(i)}$ the null hypothesis corresponding to $P_{\left(i\right)}$. Let $k=\max\{i:\;P_{\left(i\right)}\leq i\alpha /p\}$, and then reject all $H_{0,(i)}$ for i=1,⋯,k.

## 4. Simulation Study

This section examines the performance of the proposed testing procedure by a simulation study. We fix the number of factors K=3, the sample size T∈{200,400}, and let the dimensionality *p* increase from 50 to 600. Throughout the simulation, we consider testing the first column of B and repeat multiplier bootstrap 500 times.

Each row of B is generated independently from $N(0,I_{K})$, where $I_{K}$ is K×K identity matrix. Let $\mathrm{cov}\left(f_{t}\right)={\left(\sigma_{ij}^{f}\right)}_{K\times K}$ with $\sigma_{ij}^{f}=0.6^{\left|i-j\right|}$. Here, we consider two models for the covariance structure $\Sigma_{u}$.

(a) Model 1 (sparse): $\Omega_{u}={\left(\omega_{ij}\right)}_{1\leq i,j\leq p}$ where $\omega_{ii}=1$, $\omega_{ij}=0.8$ for 2(k−1)+1≤i≠j≤2k, where k=1,⋯,[p/2] and $\omega_{i,j}=0$ otherwise. $\Sigma_{u}=\Omega_{u}^{-1}$.
(b) Model 2 (non-sparse): $\Sigma_{u}={\left(\sigma_{ij}\right)}_{1\leq i,j\leq p}$ where $\sigma_{ii}=1$ and $\sigma_{ij}=0.5$ for i≠j.

Under each model, ${\left\{f_{t}\right\}}_{t=1}^{T}$ and ${\left\{u_{t}\right\}}_{t=1}^{T}$ are generated independently from $N(0,\mathrm{cov}\left(f_{t}\right))$ and $N(0,\Sigma_{u})$, respectively.

We calculate the empirical sizes of test for each column of B under each model by considering hypothesis (3) with G={1,2,⋯,p} and $b_{ik}^{\mathrm{null}}$ being the true value of $b_{ik}$. The results are summarized in Table 1. Here “NST”, “ST” denote the non-studentized, studentized Bootstrap-based test, respectively, and “EX” denotes the test using extreme value distribution. The estimated sizes of the three tests are reasonably close to the nominal level 0.05 for the values of *p* ranging from 50 to 600.

**Figure 1:**
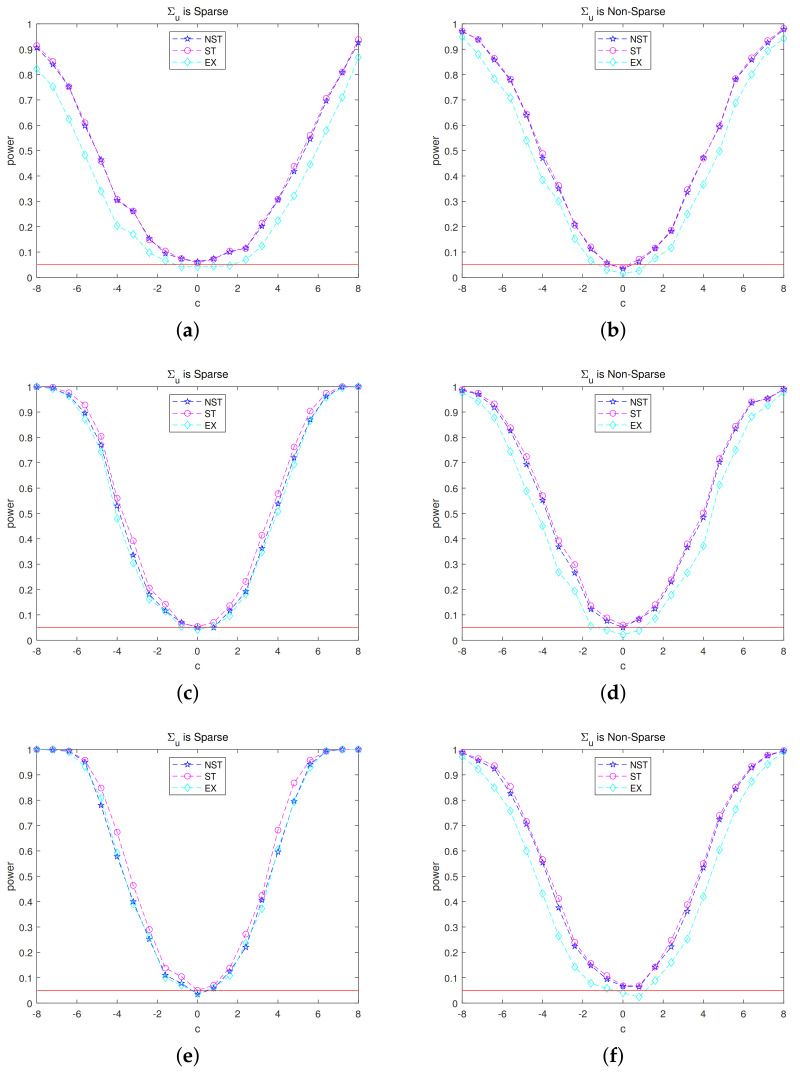
Empirical powers of the NST, ST, and EX methods. The figures in the left panels are based on Model 1, while those in the right panels are for Model 2. The red solid line corresponds to the nominal level. (**a**) p=10, (**b**) p=10, (**c**) p=200, (**d**) p=200, (**e**) p=600, (**f**) p=600.

For all i∈G, by varying $b_{ik}=b_{ik}^{\mathrm{null}}+c/40$ with c=±0.8ℓ and ℓ=0,…,10, we plot the empirical powers of $M_{T,k}$ and $M_{T,k}^{*}$ in Figure 1. For ease of presentation, we only consider p∈{10,200,600}. The results for other dimensionality are similar in spirit, and are not presented here. For all tests, the significance level is fixed at α=0.05. From Figure 1, we can tell that the empirical rejection rate grows from the nominal level to one as *c* deviates away from zero. The difference between NST test and ST test is slight. For small *p*, the EX test does not perform well because this approach requires diverging *p*. Furthermore, for non-sparse error covariance matrix, our method performs better than the EX method. These numerical results confirm our theoretical analysis.

Next, we study the numerical performance of the step-down method in Section 3 and compare it with the Bonferroni–Holm procedure. Consider the following two-sided multiple testing problem; $H_{0,i}:b_{ij}={\tilde{b}}_{ij}^{\mathrm{null}}$ among all i=1,2,⋯,p with j=1. For Models 1 and 2, the first $s_{0}$ entries of ${\left\{{\tilde{b}}_{ij}^{\mathrm{null}}\right\}}_{i=1}^{p}$ are $b_{ij}^{\mathrm{null}}+0.5$ and $b_{ij}^{\mathrm{null}}+0.35$, respectively, and the rest are equal to $b_{ij}^{\mathrm{null}}$. We set T∈{200,400} and p∈{50,200,500,600}.

We employ both the step-down method based on the studentized/non-studentized test statistic, and the Bonferroni–Holm procedure (based on the studentized test statistic) to control the FWER. We denote these three procedures by NST-FWER, ST-FWER, and BH-FWER, respectively. For comparison, we also consider using Benjamini–Hochberg procedure to control FDR. We denote this procedure by BH-FDR. Based on 500 replications, we calculate the average empirical FWER

$$\;\mathrm{Average}\;\{I\left\{\;\mathrm{At}\;\mathrm{least}\;\mathrm{one}\;\mathrm{hypothesis}\;H_{0,i}\;\mathrm{is}\;\mathrm{rejected}\;,i\in \left\{s_{0}+1,\cdots ,p\right\}\right\}\}$$

for methods NST-FWER, ST-FWER, and BH-FWER, the average empirical FDR

$$\;\mathrm{Average}\;\left\{\sum_{i\in S_{0}}I\left\{H_{0,i}\;\mathrm{is}\;\mathrm{rejected}\right\}/\sum_{i\in G}I\left\{H_{0,i}\;\mathrm{is}\;\mathrm{rejected}\right\}\right\}$$

for method BH-FDR, and the average empirical power

$$\;\mathrm{Average}\;\left\{\sum_{i\in S_{0}}I\left\{H_{0,i}\;\mathrm{is}\;\mathrm{rejected}\right\}/s_{0}\right\}$$

for all the four methods, where $S_{0}=\left\{1,2,\cdots ,s_{0}\right\}$ and G={1,⋯,p}. Under each model, we consider the case $s_{0}=3$ and $s_{0}=15$. Table 2 and Table 3 report the empirical FWER, FDR, and the average power. From Table 2 and Table 3, the proposed and Bonferroni–Holm procedures provide similar control on the FWER, and Benjamini–Hochberg procedure can control FDR. The empirical powers of the step-down method and Benjamini–Hochberg procedure are higher than that of the Bonferroni–Holm procedure. It is also seen that controlling the FDR is more powerful than controlling the FWER.

## 5. Real Data Analysis

This section conducts hypothesis testing for financial data from 1 January 2017 to 14 March 2018. The dataset consists of daily returns of 491 stocks from S&P 500 index. In addition, we collected Fama–French three factors [21] in the same period. In summary, the panel matrix is a 300 by 491 matrix Y, in addition to a factor matrix F of size 300 by 3. Here, 300 is the number of days and 491 is the number of stocks.

We first centralize and standardize the factor matrix F and Y is centralized as well. We consider testing the sparsity of each column of B and repeat the multiplier bootstrap 500 times. Simultaneous test of parameters corresponding to multiple factors is also considered. The hypotheses are

$$H_{0}:b_{ik}=0\;\;\mathrm{for}\;\mathrm{all}\;\left(i,k\right)\in s\;\mathrm{versus}\;H_{1}:b_{ik}\neq 0\;\mathrm{for}\;\mathrm{some}\;(i,k)\in s,$$

where $s=\{(i,k):k\in s_{*}\subset \left\{1,2,3\right\},\;|{\hat{b}}_{ik}|\;\mathrm{are}\;\mathrm{the}\;\mathrm{smallest}\;\beta \%\;\mathrm{among}\;\left\{\right|{\hat{b}}_{ik}{\left|\right\}}_{i\in \left\{1,\ldots ,p\right\};k\in s_{*}}\}$, with $s_{*}=\left\{1\right\},\left\{2\right\},\left\{3\right\}$ or {2,3} and β=10,30,50,70,90. The results are depicted in Table 4. For the first column of B, it is therefore not reasonable to assume $b_{i1}=0$. However, we can claim that the last two columns of B are sparse. Hence, a sufficiently large number of stocks are not influenced by the last two factors.

## Figures and Tables

**Table 1:** Empirical sizes of tests, α=0.05, T=400, and 500 replications.

	*p* = 50	*p* = 100	*p* = 200	*p* = 400	*p* = 600
Model 1					
NST	0.076	0.060	0.058	0.050	0.058
ST	0.074	0.064	0.064	0.058	0.078
EX	0.046	0.046	0.038	0.046	0.058
Model 2					
NST	0.050	0.052	0.056	0.060	0.038
ST	0.070	0.058	0.064	0.068	0.048
EX	0.038	0.030	0.024	0.018	0.016

**Table 2:** Empirical family-wise error rate (FWER) and false discovery rate (FDR) with power in the brackets of multiple testing based on Model 1, α=0.05, and 500 replications.

*T*	$s_{0}$	Method	*p* = 50	*p* = 200	*p* = 500	*p* = 600
200	3	NST-FWER	0.058 (0.551)	0.062 (0.405)	0.048 (0.309)	0.056 (0.291)
		ST-FWER	0.074 (0.554)	0.082 (0.431)	0.086 (0.337)	0.090 (0.324)
		BH-FWER	0.054 (0.528)	0.070 (0.409)	0.074 (0.319)	0.068 (0.300)
		BH-FDR	0.061 (0.635)	0.064 (0.470)	0.086 (0.380)	0.069 (0.353)
	15	NST-FWER	0.056 (0.569)	0.050 (0.412)	0.040 (0.306)	0.046 (0.303)
		ST-FWER	0.066 (0.583)	0.086 (0.430)	0.074 (0.334)	0.084 (0.327)
		BH-FWER	0.060 (0.561)	0.066 (0.410)	0.056 (0.310)	0.068 (0.309)
		BH-FDR	0.043 (0.810)	0.064 (0.655)	0.06 (0.518)	0.061 (0.509)
400	3	NST-FWER	0.050 (0.935)	0.058 (0.889)	0.062 (0.839)	0.058 (0.808)
		ST-FWER	0.070 (0.937)	0.062 (0.885)	0.078 (0.842)	0.066 (0.813)
		BH-FWER	0.052 (0.931)	0.054 (0.873)	0.062 (0.834)	0.052 (0.795)
		BH-FDR	0.057 (0.957)	0.056 (0.924)	0.064 (0.889)	0.068 (0.863)
	15	NST-FWER	0.058 (0.947)	0.054 (0.881)	0.040 (0.819)	0.070 (0.815)
		ST-FWER	0.052 (0.946)	0.066 (0.881)	0.058 (0.825)	0.084 (0.882)
		BH-FWER	0.050 (0.942)	0.056 (0.871)	0.050 (0.809)	0.060 (0.806)
		BH-FDR	0.035 (0.989)	0.052 (0.968)	0.056 (0.946)	0.059 (0.941)

**Table 3:** Empirical FWER and FDR with power in the brackets of multiple testing based on Model 2, α=0.05, and 500 replications.

*T*	$s_{0}$	Method	*p* = 50	*p* = 200	*p* = 500	*p* = 600
200	3	NST-FWER	0.044 (0.805)	0.052 (0.692)	0.066 (0.622)	0.056 (0.609)
		ST-FWER	0.058 (0.807)	0.066 (0.701)	0.084 (0.638)	0.066 (0.621)
		BH-FWER	0.030 (0.759)	0.042 (0.620)	0.024 (0.517)	0.024 (0.505)
		BH-FDR	0.039 (0.819)	0.046 (0.691)	0.038 (0.592)	0.030 (0.570)
	15	NST-FWER	0.042 (0.805)	0.060 (0.697)	0.058 (0.626)	0.048 (0.618)
		ST-FWER	0.050 (0.809)	0.080 (0.708)	0.080 (0.637)	0.072 (0.630)
		BH-FWER	0.028 (0.757)	0.042 (0.621)	0.034 (0.530)	0.038 (0.519)
		BH-FDR	0.035 (0.922)	0.044 (0.822)	0.046 (0.746)	0.040 (0.717)
400	3	NST-FWER	0.060 (0.989)	0.052 (0.985)	0.052 (0.971)	0.050 (0.970)
		ST-FWER	0.064 (0.989)	0.054 (0.985)	0.068 (0.970)	0.072 (0.973)
		BH-FWER	0.046 (0.983)	0.022 (0.975)	0.026 (0.951)	0.024 (0.945)
		BH-FDR	0.045 (0.995)	0.034 (0.990)	0.040 (0.972)	0.035 (0.973)
	15	NST-FWER	0.066 (0.992)	0.072 (0.986)	0.056 (0.975)	0.056 (0.975)
		ST-FWER	0.072 (0.992)	0.076 (0.986)	0.056 (0.975)	0.064 (0.975)
		BH-FWER	0.046 (0.988)	0.036 (0.973)	0.024 (0.952)	0.022 (0.950)
		BH-FDR	0.043 (0.999)	0.042 (0.998)	0.031 (0.993)	0.044 (0.992)

**Table 4:** Results of sparse testing.

	β = 10	β = 30	β = 50	β = 70	β = 90
1st loading	R	R	R	R	R
2nd loading	A	A	A	A	A
3rd loading	A	A	A	A	R
2nd and 3rd loading	A	A	A	R	R

Note: “A” means accepting the null hypothesis; “R” denotes rejecting the null hypothesis.

## Appendix A. Technical Details

We prove the main results in this section. First, we introduce some notations. Throughout this section, we denote by $c,c^{\prime },C,C^{\prime },C_{i}$ constants that do not depend on p,T and may vary from place to place. Define $T_{0}=\max_{i\in G}\sum_{t=1}^{T}\xi_{it}/\sqrt{T}$ and $T_{1}=\max_{i\in G}\sum_{t=1}^{T}{\hat{\xi }}_{it}/\sqrt{T}$. Let ${\left\{z_{t}\right\}}_{t=1}^{T}$ be a sequence of $N(0,\Omega_{f}\left(k,k\right)\Sigma_{u})$ vectors. Define $c_{W_{0}}\left(\alpha \right)=\inf\left\{t\in R:P\left(W_{0}\leq t|\left(Y,F\right)\right)\geq 1-\alpha \right\}$ with $W_{0}=\max_{i\in G}T^{-1/2}\sqrt{{\hat{\Omega }}_{f}\left(k,k\right)}\sum_{t=1}^{T}{\hat{u}}_{it}e_{t}$ and $c_{Z_{0}}\left(\alpha \right)=\inf\left\{t\in R:P\left(Z_{0}\leq t|\left(Y,F\right)\right)\geq 1-\alpha \right\}$ with $Z_{0}=\max_{i\in G}\sum_{t=1}^{T}z_{it}/\sqrt{T}$. Denote by $\Sigma_{f}\left(m,n\right)=v_{m}^{\prime }\Sigma_{f}v_{n}$ with $\Sigma_{f}=\Omega_{f}^{-1}$. We begin by presenting some useful lemmas that will be used in the proofs of the main results.

**Lemma** **A1.**
*Suppose that the random variables $Z_{1},Z_{2}$ both satisfy the exponential-type tail condition: There exist $r_{1},r_{2}\in \left(0,1\right)$ and $b_{1},b_{2}>0$ such that ∀s>0,*

$$P(|Z_{i}\left|>s\right)\leq \exp\left\{1-{\left(s/b_{i}\right)}^{r_{i}}\right\},\;i=1,2.$$

*Then, for some $r_{3}$ and $b_{3}>0$, and any s>0,*

(i) *$P(|Z_{1}Z_{2}\left|>s\right)\leq \exp\left\{1-{\left(s/b_{3}\right)}^{r_{3}}\right\}$, where $r_{3}\in \left(0,r\right)$ with $r=r_{1}r_{2}/\left(r_{1}+r_{2}\right)$,*
(ii) *$P\left(|Z|>s\right)\leq \exp\left\{1-{\left(s/b_{3}\right)}^{r_{3}}\right\}$, where $Z=\max\{Z_{1},Z_{2}\}$.*

**Proof of Lemma A1.** The proof of the first claim can be found in the proof of Lemma A.2 in [4], thus we prove the second claim. For any s>0, we have

$$\begin{array}{cc} P(|Z|>s) & \leq P(|Z_{1}\left|>s\right)+P(|Z_{2}\left|>s\right)\\ & =\exp\left\{1-{\left(s/b_{1}\right)}^{r_{1}}\right\}+\exp\left\{1-{\left(s/b_{2}\right)}^{r_{2}}\right\}\\ & \leq 2\exp\{1-{\left(s/b\right)}^{r}\}, \end{array}$$

where $b^{r}=\max\left\{b_{1}^{r_{1}},b_{2}^{r_{2}}\right\}$. Pick up an $r_{3}\in \left(0,r\right)$, and $b_{3}>\max\left\{{\left(r_{3}/r\right)}^{1/r}b,{\left(1+\log2\right)}^{1/r}b\right\}$; then, it can be shown that $F\left(s\right)={\left(s/b\right)}^{r}-{\left(s/b_{3}\right)}^{r_{3}}$ is increasing when $s>b_{3}$. Therefore, $F\left(s\right)>F\left(b_{3}\right)>\log2$ when $s>b_{3}$, which implies when $s>b_{3}$,

$$P\left(|Z|>s\right)\leq 2\exp\left\{1-{\left(s/b\right)}^{r}\right\}\leq \exp\left\{1-{\left(s/b_{3}\right)}^{r_{3}}\right\}.$$

When $s\leq b_{3}$,

$$P\left(|Z|>s\right)\leq 1\leq \exp\left\{1-{\left(s/b_{3}\right)}^{r_{3}}\right\}.$$

Then the proof is complete. ☐

**Lemma** **A2.**
*Under Assumptions 1–4, we have*

(i) *$\max_{i,j\leq p}\left|{\hat{\sigma }}_{ij}-\sigma_{ij}\right|=O_{p}\left(\sqrt{\log p/T}\right)$.*
(ii) *$\max_{i\leq p,k\leq K}\left|\left(1/T\right)\sum_{t=1}^{T}f_{kt}u_{it}\right|=O_{p}\left(\sqrt{\log p/T}\right)$.*
(iii) *$\max_{i,j\leq K}\left|\left(1/T\right)\sum_{t=1}^{T}f_{it}f_{jt}-Ef_{it}f_{jt}\right|=O_{p}\left(\sqrt{\log T/T}\right)$.*

**Proof of Lemma A2.** For a proof, see the proof of Lemma A.3 and Lemma B.1 in [4]. ☐

**Lemma** **A3.**
*If a random variable X satisfies exponential-type tail: there exist r>0 and b>0 such that ∀s>0, $P\left(|X|>s\right)\leq \exp\left\{1-{\left(s/b\right)}^{r}\right\}$. Then E(|X|)=O(1).*

**Proof of Lemma A3.** Note that

$$\begin{array}{cc} E(|X|) & =\int_{0}^{\infty }P\left(|X|>x\right)dx\\ & \leq \int_{0}^{\infty }\exp\left\{1-{\left(x/b\right)}^{r}\right\}dx\\ & ≲\int_{0}^{\infty }\exp\left(-x^{r}\right)dx:=I \end{array}$$

It is not hard to check when r≥1, $I\leq 1+e^{-1}$. When r<1, I=αΓ(α), where α=1/r and $\Gamma \left(\alpha \right)=\int_{0}^{\infty }e^{-x}x^{\alpha -1}dx=O\left(1\right)$. Then, the proof is complete. ☐

**Lemma** **A4.**
*Under the assumptions in Theorem 1, there exist constants c,C>0 such that*

$$\rho :=\underset{t\in R}{\sup}\left|P\left(T_{0}\leq t\right)-P\left(Z_{0}\leq t\right)\right|\leq CT^{-c}.$$

**Proof of Lemma A4.** Implied by Assumption 3, we have ${\left(\log\left(pT\right)\right)}^{7}/T\leq C_{1}T^{-c_{1}}$ for some constants $c_{1},C_{1}>0$. We then apply Corollary 2.1 of [25] to the sequence $\xi_{it}$. What we should check is its Condition (E.2), that is, uniformly over *i*,

$$c_{0}\leq E{\left(\xi_{it}\right)}^{2}\leq C_{0},\;\max_{k=1,2}E(|\xi_{it}{|}^{k+2}/B^{k})+E\{(\max_{1\leq i\leq p}|\xi_{it}{\left|/B\right)}^{4}\}\leq 4,$$

where $c_{0},C_{0}>0$ and *B* is some large enough constant. By Lemmas A1 and A3 we have $E\left(f_{it}f_{jt}\right)=O\left(1\right)$ uniformly for i,j≤K. This implies $∥\Omega_{f}{∥}_{\infty }=O\left(1\right)$. Uniformly for i≤p, we have

$$\xi_{it}=u_{it}f_{t}^{\prime }\Omega_{f}v_{k}\leq u_{it}∥f_{t}{∥}_{\infty }{∥\Omega_{f}v_{k}∥}_{1}:=\gamma_{it}.$$

By Lemma A1, we know $\gamma_{it}$ and $\max_{1\leq i\leq p}\left|\gamma_{it}\right|$ is exponential-type tail. Then by Lemma A3 we have $E(|\xi_{it}{|}^{4})\leq E(|\gamma_{it}{|}^{4})$=O(1) and $E(\max_{i\leq p}|\xi_{it}{\left|\right)}^{4}\leq E(\max_{i\leq p}|\gamma_{it}{\left|\right)}^{4}=O\left(1\right)$. Thus, we can find large enough *B* such that the above condition is satisfied. Then, the proof is complete. ☐

**Lemma** **A5.**
*Under the assumptions in Theorem 1, there exists a sequence of positive numbers $\alpha_{T}\to \infty$ such that $\alpha_{T}/p=o\left(1\right)$ and $P(\alpha_{T}{\left(\log p\right)}^{2}|{\hat{\Omega }}_{f}\left(k,k\right)-\Omega_{f}\left(k,k\right)|>1)\to 0$.*

**Proof of Lemma A5.** By Lemma A2 (iii), we have $∥{\hat{\Omega }}_{f}^{-1}-\Omega_{f}^{-1}{∥}_{\infty }=O_{p}\left(\sqrt{\log T/T}\right)$. Since $∥\Omega_{f}∥=O\left(1\right)$ and $∥{\hat{\Omega }}_{f}{∥}_{\infty }=O_{p}\left(1\right)$, we have

$$\begin{array}{cc} ∥{\hat{\Omega }}_{f}-\Omega_{f}{∥}_{\infty } & =∥{\hat{\Omega }}_{f}\left(\Omega_{f}^{-1}-{\hat{\Omega }}_{f}^{-1}\right)\Omega_{f}{∥}_{\infty }\\ & \leq ∥{\hat{\Omega }}_{f}{∥}_{\infty }∥{\hat{\Omega }}_{f}^{-1}-\Omega_{f}^{-1}{∥}_{\infty }{∥\Omega_{f}∥}_{\infty }\\ & =O_{p}\left(\sqrt{\log T/T}\right). \end{array}$$

On the other hand,

$$|{\hat{\Omega }}_{f}\left(k,k\right)-\Omega_{f}\left(k,k\right)|\leq ∥{\hat{\Omega }}_{f}-\Omega_{f}{∥}_{\infty }=O_{p}\left(\sqrt{\log T/T}\right).$$

Choosing $\alpha_{T}=\sqrt{\log T}$, by Assumption 3, the proof is complete. ☐

**Lemma** **A6.**
*Under the assumptions in Theorem 1, we have for every α∈(0,1) and ϑ>0,*

$$\begin{array}{cc} & P(c_{W_{0}}\left(\alpha \right)\leq c_{Z_{0}}\left(\alpha +\pi \left(\vartheta \right)\right))\geq 1-P\left(\Delta >\vartheta \right),\\ & P(c_{Z_{0}}\left(\alpha \right)\leq c_{W_{0}}\left(\alpha +\pi \left(\vartheta \right)\right))\geq 1-P\left(\Delta >\vartheta \right), \end{array}$$

**Proof of Lemma A6.** For ϑ>0, let $\pi \left(\vartheta \right)=C_{2}\vartheta^{1/3}{\left(1\vee \log\left(p/\vartheta \right)\right)}^{2/3}$ with $C_{2}>0$. Recall that $\Delta =\max_{1\leq i,j\leq p}\left|{\hat{\Omega }}_{f}\left(k,k\right){\hat{\sigma }}_{ij}-\Omega_{f}\left(k,k\right)\sigma_{ij}\right|$. As $|\Omega_{f}\left(k,k\right)|=O\left(1\right)$ uniformly for k≤K, by Lemma A2 (i), we have

$$\Delta =O_{p}\left(\right|{\hat{\Omega }}_{f}\left(k,k\right)-\Omega_{f}\left(k,k\right)\left|+\sqrt{(\log p)/T}\right).$$

By Lemma A5 and Assumption 3, choosing $\vartheta =1/(\alpha_{T}{\left(\log p\right)}^{2})$, we have P(Δ>ϑ)=o(1). By Lemma 3.1 of [25], on the event {(Y,F):Δ≤ϑ}, we have $|P\left(Z_{0}\leq t\right)-P\left(P\left(W_{0}\leq t|\left(Y,F\right)\right)\right|\leq \pi \left(\vartheta \right)$ for all t∈R, and so on this event

$$\begin{array}{cc} P(P(W_{0}\leq c_{Z_{0}}\left(\alpha +\pi \left(\vartheta \right)\right)|\left(Y,F\right))) & \geq P(Z_{0}\leq c_{Z_{0}}\left(\alpha +\pi \left(\vartheta \right)\right))-\pi \left(\vartheta \right)\\ & \geq \alpha +\pi (\vartheta )-\pi (\vartheta )=\alpha , \end{array}$$

implying the first claim. The second claim follows similarly. ☐

**Lemma** **A7.**
*Under the assumptions in Theorem 1, there exist $\zeta_{1},\zeta_{2}>0$ such that*

$$P\left(|\max_{1\leq i\leq p}\sum_{t=1}^{T}{\hat{\xi }}_{it}/\sqrt{T}-\max_{1\leq i\leq p}\sum_{t=1}^{T}\xi_{it}/\sqrt{T}|>\zeta_{1}\right)<\zeta_{2},$$

*where $\zeta_{1}\sqrt{1\vee \log(p/\zeta_{1})}=o\left(1\right)$, $\zeta_{2}=o\left(1\right)$.*

**Proof of Lemma A7.** The arguments in the proof of Lemma A5 imply that

$$∥{\hat{\Omega }}_{f}v_{k}-\Omega_{f}v_{k}{∥}_{1}=O_{p}\left(\sqrt{\log T/T}\right).$$

By Lemma A2 (ii), uniformly for i≤p, we have

$$\begin{array}{cc} \left|\sum_{t=1}^{T}{\hat{\xi }}_{it}/\sqrt{T}-\sum_{t=1}^{T}\xi_{it}/\sqrt{T}\right| & =|\sum_{t=1}^{T}u_{it}f_{t}^{\prime }\left({\hat{\Omega }}_{f}v_{k}-\Omega_{f}v_{k}\right)/\sqrt{T}|\\ & \leq ∥{\hat{\Omega }}_{f}v_{k}-\Omega_{f}v_{k}{∥}_{1}\max_{i\leq p,k\leq K}|\frac{1}{\sqrt{T}}\sum_{t=1}^{T}f_{kt}u_{it}|\\ & =O_{p}\left(\sqrt{\log p\log T/T}\right). \end{array}$$

Choosing $\zeta_{1}^{2}=O\left(\sqrt{\log p\log T/T}\right)$, we have

$$P\left(\max_{1\leq i\leq p}|\sum_{t=1}^{T}{\hat{\xi }}_{it}/\sqrt{T}-\sum_{t=1}^{T}\xi_{it}/\sqrt{T}|>\zeta_{1}\right)\leq \zeta_{2},\;\zeta_{2}=o\left(1\right).$$

Note that

$$|\max_{1\leq i\leq p}\sum_{t=1}^{T}{\hat{\xi }}_{it}/\sqrt{T}-\max_{1\leq i\leq p}\sum_{t=1}^{T}\xi_{it}/\sqrt{T}|\leq \max_{1\leq i\leq p}|\sum_{t=1}^{T}{\hat{\xi }}_{it}/\sqrt{T}-\sum_{t=1}^{T}\xi_{it}/\sqrt{T}|,$$

then the proof is complete. ☐

**Lemma** **A8.**
*Under the assumptions in Theorem 4, we have*

(i) *$\max_{i,j,m,n}\left|\left(1/T\right)\sum_{t=1}^{T}u_{it}u_{jt}f_{mt}f_{nt}-\sigma_{ij}\Sigma_{f}\left(m,n\right)\right|=O_{p}\left(\sqrt{\log p/T}\right)$,*
(ii) *$\max_{i,j,m,n}\left|\left(1/T\right)\sum_{t=1}^{T}u_{it}f_{jt}f_{mt}f_{nt}\right|=O_{p}\left(\sqrt{\log p/T}\right)$,*

**Proof of Lemma A8.** (i) By Assumption 1 and Lemma A1, $u_{it}f_{mt}$ satisfies the exponential tail condition, with parameter $2r_{1}r_{2}/\left(3r_{1}+3r_{2}\right)$ as shown in Lemma A1. Thus, $u_{it}u_{jt}f_{mt}f_{nt}$ satisfies the exponential tail condition, with parameter $r_{1}r_{2}/\left(4r_{1}+4r_{2}\right)$. It follows from $1.5(r_{1}^{-1}+r_{2}^{-1})>1$ that $\gamma_{3}<1$. Therefore, by the Bernstein’s inequality [23], there exist constants $C_{i}$, i=1,⋯,5, for any s>0

(A1) $$\begin{array}{cc} \; & \max_{i,j,m,n}P\left(|\frac{1}{T}\sum_{t=1}^{T}u_{it}u_{jt}f_{mt}f_{nt}-\sigma_{ij}\Sigma_{f}\left(m,n\right)|\geq s\right)\leq T\exp\left(-\frac{{\left(Ts\right)}^{\gamma_{3}}}{C_{1}}\right)\\ \; & +\exp\left(-\frac{T^{2}s^{2}}{C_{2}\left(1+TC_{3}\right)}\right)+\exp\left(-\frac{{\left(Ts\right)}^{2}}{C_{4}T}\exp\left(\frac{{\left(Ts\right)}^{\gamma_{3}\left(1-\gamma_{3}\right)}}{C_{5}{\left(\log Ts\right)}^{\gamma_{3}}}\right)\right). \end{array}$$

Using Bonferroni’s method, we have

$$\begin{array}{cc} P( & \max_{i,j,m,n}|\frac{1}{T}\sum_{t=1}^{T}u_{it}u_{jt}f_{mt}f_{nt}-\sigma_{ij}\Sigma_{f}\left(m,n\right)|>s)\\ & \leq {\left(pK\right)}^{2}\max_{i,j,m,n}P\left(|\frac{1}{T}\sum_{t=1}^{T}u_{it}u_{jt}f_{mt}f_{nt}-\sigma_{ij}\Sigma_{f}\left(m,n\right)|>s\right). \end{array}$$

Let $s=C\sqrt{(\log p)/T}$ for some C>0. It is not hard to check that when ${\left(\log p\right)}^{2/\gamma_{3}-1}=o\left(T\right)$ (by assumption), for large enough *C*,

$$p^{2}T\exp\left(-\frac{{\left(Ts\right)}^{\gamma_{3}}}{C_{1}}\right)+p^{2}\exp\left(-\frac{{\left(Ts\right)}^{2}}{C_{4}T}\exp\left(\frac{{\left(Ts\right)}^{\gamma_{3}\left(1-\gamma_{3}\right)}}{C_{5}{\left(\log Ts\right)}^{\gamma_{3}}}\right)\right)=o\left(\frac{1}{p^{2}}\right)$$

and

$$p^{2}\exp\left(-\frac{T^{2}s^{2}}{C_{2}\left(1+TC_{3}\right)}\right)=O\left(\frac{1}{p^{2}}\right).$$

As K=O(1), this proves (i). (ii) By Assumption 1 and Lemma A1, $u_{it}f_{jt}f_{mt}f_{nt}$ satisfies the exponential tail condition for the tail parameter $r_{1}r_{2}/\left(9r_{1}+3r_{2}\right)$. Therefore, again by the Bernstein’s inequality and the Bonferroni method on $u_{it}f_{jt}f_{mt}f_{nt}$ similar to (A1) with the parameter $r_{3}^{-1}=3r_{1}^{-1}+9r_{2}^{-1}$, it follows from $1.5(r_{1}^{-1}+r_{2}^{-1})>1$ that $r_{3}<1$. Thus, when $s=C\sqrt{\log p/T}$ for large enough *C*, as *K* is fixed, the term

$$pK^{3}\exp\left(-\frac{T^{2}s^{2}}{C_{2}\left(1+TC_{3}\right)}\right)\leq p^{-2},$$

and the rest terms on the right-hand side of the inequality, multiplied by $pK^{3}$ are of order $o(p^{-2})$. Hence when ${\left(\log p\right)}^{2/r_{3}-1}=o\left(T\right)$ (by assumption), we have

$$\max_{i,j,m,n}|\frac{1}{T}\sum_{t=1}^{T}u_{it}f_{jt}f_{mt}f_{nt}|=O_{p}\left(\sqrt{\log p/T}\right),$$

which completes the proof. ☐

**Lemma** **A9.**
*Under the assumptions in Theorem 4, we have*

(i) $\max_{i,j,m,n}\left|\left(1/T\right)\sum_{t=1}^{T}\left({\hat{u}}_{it}{\hat{u}}_{jt}f_{mt}f_{nt}-u_{it}u_{jt}f_{mt}f_{nt}\right)\right|=O_{p}\left(\log p/T\right).$
(ii) $\max_{i,j,m,n}\left|\left(1/T\right)\sum_{t=1}^{T}{\hat{u}}_{it}{\hat{u}}_{jt}f_{mt}f_{nt}-\sigma_{ij}\Sigma_{f}\left(m,n\right)\right|=O_{p}\left(\sqrt{\log p/T}\right).$

**Proof of Lemma A9.** (i) By the triangular inequality, we have

$$\begin{array}{c} \left|\frac{1}{T}\sum_{t=1}^{T}\left({\hat{u}}_{it}{\hat{u}}_{jt}f_{mt}f_{nt}-u_{it}u_{jt}f_{mt}f_{nt}\right)\right|\\ \leq \underset{I}{\underset{︸}{\left|\frac{1}{T}\sum_{t=1}^{T}\left({\hat{u}}_{it}{\hat{u}}_{jt}f_{mt}f_{nt}-{\hat{u}}_{it}u_{jt}f_{mt}f_{nt}\right)\right|}}+\underset{II}{\underset{︸}{\left|\frac{1}{T}\sum_{t=1}^{T}\left({\hat{u}}_{it}u_{jt}f_{mt}f_{nt}-u_{it}u_{jt}f_{mt}f_{nt}\right)\right|}} \end{array}$$

For *I*, we have

$$\begin{array}{cc} I & =|\frac{1}{T}\sum_{t=1}^{T}{\hat{u}}_{it}{\left({\hat{b}}_{j}-b_{j}\right)}^{\prime }f_{t}f_{mt}f_{nt}|\\ & \leq \underset{i}{\underset{︸}{\left|\frac{1}{T}\sum_{t=1}^{T}{\left({\hat{b}}_{i}-b_{i}\right)}^{\prime }f_{t}{\left({\hat{b}}_{j}-b_{j}\right)}^{\prime }f_{t}f_{mt}f_{nt}\right|}}+\underset{ii}{\underset{︸}{\left|\frac{1}{T}\sum_{t=1}^{T}u_{it}{\left({\hat{b}}_{j}-b_{j}\right)}^{\prime }f_{t}f_{mt}f_{nt}\right|}} \end{array}$$

By Lemma 3.1 of [4], we have $\max_{i\leq p}∥{\hat{b}}_{i}-b_{i}∥=O_{p}\left(\sqrt{\log p/T}\right)$. It is straightforward to see that

$$\max_{m,n\leq K}∥\frac{1}{T}\sum_{t=1}^{T}f_{t}f_{t}^{\prime }f_{mt}f_{nt}{∥}_{\infty }=O_{p}\left(1\right).$$

then we have,

$$i\leq \max_{i,m,n}{∥{\hat{b}}_{i}-b_{i}∥}^{2}∥\frac{1}{T}\sum_{t=1}^{T}f_{t}f_{t}^{\prime }f_{mt}f_{nt}{∥}_{\infty }=O_{p}\left(\log p/T\right).$$

By Lemma A8 (ii), we have $∥\left(1/T\right)\sum_{t=1}^{T}u_{it}f_{t}f_{mt}f_{nt}{∥}_{\infty }=O_{p}\left(\sqrt{\log p/T}\right)$, which implies that

$$ii\leq \max_{j\leq p}∥{\hat{b}}_{j}-b_{j}∥∥\frac{1}{T}\sum_{t=1}^{T}u_{it}f_{t}f_{mt}f_{nt}{∥}_{\infty }=O_{p}\left(\log p/T\right)$$

Part II is similar to ii, thus we have

$$II=|\frac{1}{T}\sum_{t=1}^{T}{\left({\hat{b}}_{i}-b_{i}\right)}^{\prime }f_{t}u_{jt}f_{mt}f_{nt}|\leq O_{p}\left(\log p/T\right).$$

Then the proof is complete. (ii) By the triangular inequality, we have

$$\begin{array}{c} \max_{i,j,m,n}|\frac{1}{T}\sum_{t=1}^{T}{\hat{u}}_{it}{\hat{u}}_{jt}f_{mt}f_{nt}-\sigma_{ij}\Sigma_{f}\left(m,n\right)|\\ \leq \max_{i,j,m,n}|\frac{1}{T}\sum_{t=1}^{T}\left({\hat{u}}_{it}{\hat{u}}_{jt}f_{mt}f_{nt}-u_{it}u_{jt}f_{mt}f_{nt}\right)|+\max_{i,j,m,n}|\frac{1}{T}\sum_{t=1}^{T}u_{it}u_{jt}f_{mt}f_{nt}-\sigma_{ij}\Sigma_{f}\left(m,n\right)|\\ =O_{p}\left(\sqrt{\log p/T}\right), \end{array}$$

which proves the result. ☐

**Proof of Theorem 1.** Without loss of generality, we set G={1,2,⋯,p}. First, we prove the following fact,

(A2) $$\underset{\alpha \in (0,1)}{\sup}\left|P\left(T_{1}>c_{W_{0}}\left(\alpha \right)\right)-\alpha \right|=o\left(1\right),$$

For ϑ>0, let $\pi \left(\vartheta \right):=C_{2}\vartheta^{1/3}{\left(1\vee \log\left(p/\vartheta \right)\right)}^{2/3}$ with $C_{2}>0$. In addition, Let $\kappa_{1}\left(\vartheta \right):=c_{Z_{0}}\left(\alpha -\zeta_{2}-\pi \left(\vartheta \right)\right)$ and $\kappa_{2}\left(\vartheta \right):=c_{Z_{0}}\left(\alpha +\zeta_{2}+\pi \left(\vartheta \right)\right)$. For every α∈(0,1), note that

$$\begin{array}{cc} & \;\;P(\left\{T_{1}\leq c_{W_{0}}\left(\alpha \right)\right\}⊖\left\{T_{0}\leq c_{Z_{0}}\left(\alpha \right)\right\})\\ & {≲}_{\left(1\right)}P\left(\kappa_{1}\left(\vartheta \right)-2\zeta_{1}<T_{0}\leq \kappa_{2}\left(\vartheta \right)+2\zeta_{1}\right)+P\left(\Delta >\vartheta \right)+\zeta_{2}\\ & {≲}_{\left(2\right)}P\left(\kappa_{1}\left(\vartheta \right)-2\zeta_{1}<Z_{0}\leq \kappa_{2}\left(\vartheta \right)+2\zeta_{1}\right)+P\left(\Delta >\vartheta \right)+\rho +\zeta_{2}\\ & {≲}_{\left(3\right)}\pi \left(\vartheta \right)+P\left(\Delta >\vartheta \right)+\rho +\zeta_{1}\sqrt{1\vee \log(p/\zeta_{1})}+\zeta_{2}, \end{array}$$

where (1) follows from Lemmas A6 and A7, (2) follows from Lemma A4, and (3) follows from Lemma 2.1 in [25] and the fact that $Z_{0}$ has no point masses. Then, by the definition of ρ in Lemma A4, we have

$$\underset{\alpha \in (0,1)}{\sup}\left|P\left(T_{1}>c_{W_{0}}\left(\alpha \right)\right)-\alpha \right|\leq \rho_{⊖}+\rho ,$$

where $\rho_{⊖}=\sup_{\alpha \in (0,1)}P\left(\left\{T_{1}\leq c_{W_{0}}\left(\alpha \right)\right\}⊖\left\{T_{0}\leq c_{Z_{0}}\left(\alpha \right)\right\}\right)$. The right-hand side of the above inequality is o(1), which has proved (A2). Since $\max_{i\in G}\sqrt{T}\left|{\hat{b}}_{ik}-b_{ik}\right|=\sqrt{T}\max_{i\in G}\max\left\{{\hat{b}}_{ik}-b_{ik},b_{ik}-{\hat{b}}_{ik}\right\}$, similar arguments imply that

$$\underset{\alpha \in (0,1)}{\sup}|P\left(\max_{i\in G}\sqrt{T}\left|{\hat{b}}_{ik}-b_{ik}\right|>c_{W_{T,k}}\left(\alpha \right)\right)-\alpha |=o\left(1\right),$$

which completes the proof. ☐

**Proof of Theorem 2.** From the arguments in the proof of Lemma A6, we have

$$\Delta =O_{p}\left(\right|{\hat{\Omega }}_{f}\left(k,k\right)-\Omega_{f}\left(k,k\right)\left|+\sqrt{\log p/T}\right),$$

which implies that $\max_{1\leq i\leq p}|{\hat{\omega }}_{ii}-\omega_{ii}|=O_{p}\left(\right|{\hat{\Omega }}_{f}\left(k,k\right)-\Omega_{f}\left(k,k\right)\left|+\sqrt{\log p/T}\right)$. We then have

(A3) $$P(\omega_{ii}/2<{\hat{\omega }}_{ii}<2\omega_{ii}\;\mathrm{for}\;\mathrm{all}\;1\leq i\leq p)\to 1.$$

Define ${\bar{T}}_{1}=\max_{i\in G}\sum_{t=1}^{T}{\hat{\xi }}_{it}/\sqrt{T{\hat{\omega }}_{ii}}$ and ${\bar{T}}_{0}=\max_{i\in G}\sum_{t=1}^{T}\xi_{it}/\sqrt{T\omega_{ii}}$. Note that

$$\begin{array}{cc} & \;|{\bar{T}}_{1}-{\bar{T}}_{0}|\\ & \leq \max_{1\leq i\leq p}|\sum_{t=1}^{T}{\hat{\xi }}_{it}/\sqrt{T{\hat{\omega }}_{ii}}-\sum_{t=1}^{T}\xi_{it}/\sqrt{T\omega_{ii}}|\\ & \leq \max_{1\leq i\leq p}|\sum_{t=1}^{T}{\hat{\xi }}_{it}/\sqrt{T{\hat{\omega }}_{ii}}-\sum_{t=1}^{T}{\hat{\xi }}_{it}/\sqrt{T\omega_{ii}}|+\max_{1\leq i\leq p}|\sum_{t=1}^{T}{\hat{\xi }}_{it}/\sqrt{T\omega_{ii}}-\sum_{t=1}^{T}\xi_{it}/\sqrt{T\omega_{ii}}|\\ & \leq C^{\prime }\max_{1\leq i\leq p}|\sum_{t=1}^{T}{\hat{\xi }}_{it}/\sqrt{T}|\max_{1\leq i\leq p}\left|\sqrt{\omega_{ii}/{\hat{\omega }}_{ii}}-1\right|+C^{\prime \prime }\max_{1\leq i\leq p}|\sum_{t=1}^{T}\left({\hat{\xi }}_{it}-\xi_{it}\right)/\sqrt{T}|\\ & :=I_{1}+I_{2}, \end{array}$$

where $C^{\prime },C^{\prime \prime }>0$. On the event $\omega_{ii}/2<{\hat{\omega }}_{ii}<2\omega_{ii}$ for all 1≤i≤p,

$$\begin{array}{cc} \max_{1\leq i\leq p}\left|\sqrt{\omega_{ii}/{\hat{\omega }}_{ii}}-1\right| & \leq \max_{1\leq i\leq p}\left|\sqrt{\omega_{ii}}-\sqrt{{\hat{\omega }}_{ii}}\right|\max_{1\leq i\leq p}\sqrt{2/\omega_{ii}}\\ & \leq \max_{1\leq i\leq p}|\frac{\omega_{ii}-{\hat{\omega }}_{ii}}{\sqrt{\omega_{ii}}+\sqrt{{\hat{\omega }}_{ii}}}|\max_{1\leq i\leq p}\sqrt{2/\omega_{ii}}\\ & \leq \max_{1\leq i\leq p}\left|\omega_{ii}-{\hat{\omega }}_{ii}\right|\max_{1\leq i\leq p}1/\omega_{ii}\\ & =O_{p}\left(\right|{\hat{\Omega }}_{f}\left(k,k\right)-\Omega_{f}\left(k,k\right)\left|+\sqrt{(\log p)/T}\right). \end{array}$$

On the other hand,

$$\begin{array}{cc} \max_{1\leq i\leq p}|\sum_{t=1}^{T}{\hat{\xi }}_{it}/\sqrt{T}| & \leq \max_{1\leq i\leq p}|\sum_{t=1}^{T}\left({\hat{\xi }}_{it}-\xi_{it}\right)/\sqrt{T}|+\max_{1\leq i\leq p}|\sum_{t=1}^{T}\xi_{it}/\sqrt{T}|\\ & =O_{P}\left(\sqrt{\log p\log T/T}+\sqrt{\log p}\right)=O_{P}\left(\sqrt{\log p}\right). \end{array}$$

Therefore, on the above event, $I_{1}\leq O_{p}(\sqrt{\log p}|{\hat{\Omega }}_{f}\left(k,k\right)-\Omega_{f}\left(k,k\right)\left|+\log p/\sqrt{T}\right)$. By Lemma A5, we can find $\zeta_{1}^{\prime }$ such that $P\left(I_{1}>\zeta_{1}^{\prime }\right)=o\left(1\right)$ and $\zeta_{1}^{\prime }\sqrt{1\vee \log(p/\zeta_{1}^{\prime })}=o\left(1\right)$. Thus by Lemma A7 and (A3), we have

$$P(|{\bar{T}}_{1}-{\bar{T}}_{0}\left|>\zeta_{1}\right)\leq P\left(I_{1}+I_{2}>\zeta_{1}\right)<\zeta_{2},$$

for $\zeta_{1}\sqrt{1\vee \log(p/\zeta_{1})}=o\left(1\right)$ and $\zeta_{2}=o\left(1\right)$. Let $\bar{\Delta }=\max_{1\leq j,k\leq p}\left|\omega_{jk}/\sqrt{\omega_{jj}\omega_{kk}}-{\hat{\omega }}_{jk}/\sqrt{{\hat{\omega }}_{jj}{\hat{\omega }}_{kk}}\right|$. Note that

$$|\sqrt{\omega_{jj}\omega_{kk}}-\sqrt{{\hat{\omega }}_{jj}{\hat{\omega }}_{kk}}|=\frac{|\omega_{jj}\omega_{kk}-{\hat{\omega }}_{jj}{\hat{\omega }}_{kk}|}{\sqrt{\omega_{jj}\omega_{kk}}+\sqrt{{\hat{\omega }}_{jj}{\hat{\omega }}_{kk}}}.$$

On the event $\omega_{ii}/2<{\hat{\omega }}_{ii}<2\omega_{ii}$ for all 1≤i≤p, we have

$$\frac{|\omega_{jj}\omega_{kk}-{\hat{\omega }}_{jj}{\hat{\omega }}_{kk}|}{\sqrt{\omega_{jj}\omega_{kk}}+\sqrt{{\hat{\omega }}_{jj}{\hat{\omega }}_{kk}}}\leq \frac{|\omega_{jj}\omega_{kk}-{\hat{\omega }}_{jj}{\hat{\omega }}_{kk}|}{\sqrt{\omega_{jj}\omega_{kk}}+\sqrt{\omega_{jj}\omega_{kk}/4}}\leq \left(2/3\right)\left|\omega_{jj}\omega_{kk}-{\hat{\omega }}_{jj}{\hat{\omega }}_{kk}\right|\max_{1\leq j\leq p}1/\omega_{jj},$$

which implies that

$$\begin{array}{cc} \max_{1\leq j,k\leq p}\left|\sqrt{\omega_{jj}\omega_{kk}/{\hat{\omega }}_{jj}{\hat{\omega }}_{kk}}-1\right| & \leq \max_{1\leq j,k\leq p}\left|\sqrt{\omega_{jj}\omega_{kk}}-\sqrt{{\hat{\omega }}_{jj}{\hat{\omega }}_{kk}}\right|\max_{1\leq j\leq p}2/\omega_{jj}\\ \; & \leq \left(4/3\right)\max_{1\leq j,k\leq p}\left|\omega_{jj}\omega_{kk}-{\hat{\omega }}_{jj}{\hat{\omega }}_{kk}\right|\max_{1\leq j\leq p}1/\omega_{jj}^{2}\\ \; & =O_{p}\left(\right|{\hat{\Omega }}_{f}\left(k,k\right)-\Omega_{f}\left(k,k\right)\left|+\sqrt{(\log p)/T}\right). \end{array}$$

Choosing $\vartheta =1/(\alpha_{T}{\left(\log p\right)}^{2})$, we can show that $P\left(\bar{\Delta }>\vartheta \right)=o\left(1\right)$. The rest of the proofs are similar to those in the proof of Theorem 1. We skip the details. ☐

**Proof of Theorem 3.** Let $Z=\left(Z_{1},\cdots ,Z_{p}\right)\overset{d}{∼}N\left(0,\Theta \right)$. Following the arguments in the proof of Theorem 2, we can show that the distribution of $\max_{i\in G}\sqrt{T}\left|{\hat{b}}_{ik}-b_{ik}\right|/\sqrt{{\hat{\omega }}_{ii}}$ can be approximated by $\max_{i\in G}\left|Z_{i}\right|$. Under Assumption 5, by Lemma 6 of [15], we have for any x∈R and as |G|→∞,

$$P\left(\max_{i\in G}|Z_{i}{|}^{2}-2\log\left(|G|\right)+\log\log\left(|G|\right)\leq x\right)\to F\left(x\right):=\exp\left\{-\frac{1}{\sqrt{\pi }}\exp\left(-\frac{x}{2}\right)\right\}.$$

It implies that

(A4) $$P\left(\max_{i\in G}T|{\hat{b}}_{ik}-b_{ik}{|}^{2}/{\hat{\omega }}_{ii}\leq 2\log\left(|G|\right)-\log\log\left(|G|\right)/2\right)\to 1.$$

The bootstrap consistency result implies that

(A5) $$|{\left(c_{W_{T,k}}^{*}\left(\alpha \right)\right)}^{2}-2\log\left(|G|\right)+\log\log\left(|G|\right)-q_{\alpha }|=o_{P}\left(1\right),$$

where $q_{\alpha }$ is the 100(1−α)th quantile of F(x). Consider any i∈G such that $|b_{ik}^{\mathrm{null}}-b_{ik}|/\sqrt{\omega_{ii}}>\left(\sqrt{2}+\varepsilon_{0}\right)\sqrt{\log|G|/T}$. Using the inequality $2a_{1}a_{2}\leq \delta^{-1}a_{1}^{2}+\delta a_{2}^{2}$ for any δ>0, we have

(A6) $$T|b_{ik}^{\mathrm{null}}-b_{ik}{|}^{2}/{\hat{\omega }}_{ii}\leq \left(1+\delta^{-1}\right)T|{\hat{b}}_{ik}-b_{ik}{|}^{2}/{\hat{\omega }}_{ii}+\left(1+\delta \right)T{\left|{\hat{b}}_{ik}-b_{ik}^{\mathrm{null}}\right|}^{2}/{\hat{\omega }}_{ii},$$

where $T|{\hat{b}}_{ik}-b_{ik}{|}^{2}/{\hat{\omega }}_{ii}=o_{p}\left(\log|G|\right)$ as *i* is fixed and |G| grows. From the proof of Theorem 2, we know the difference between $T|b_{ik}^{\mathrm{null}}-b_{ik}{|}^{2}/{\hat{\omega }}_{ii}$ and $T|b_{ik}^{\mathrm{null}}-b_{ik}{|}^{2}/\omega_{ii}$ is asymptotically negligible. Thus, by (A6) and the fact that $B_{k}\in U_{G}\left(\sqrt{2}+\varepsilon_{0}\right)$, we have

(A7) $$\max_{i\in G}T{\left|{\hat{b}}_{ik}-b_{ik}^{\mathrm{null}}\right|}^{2}/{\hat{\omega }}_{ii}\geq \frac{1}{1+\delta }\left\{{\left(\sqrt{2}+\varepsilon_{0}\right)}^{2}\left(\log|G|\right)-o_{p}\left(\log|G|\right)\right\}.$$

The conclusion thus follows from (A7) and (A5) provided that δ is small enough. ☐

**Proof of Theorem 4.** Without loss of generality, we set $G^{*}=M$. Define

$$\hat{\Gamma }=\frac{1}{T}\sum_{t=1}^{T}{\hat{u}}_{it}{\hat{u}}_{jt}f_{t}f_{t}^{\prime },\;\;\mathrm{and}\;\;\Gamma =\sigma_{ij}\Sigma_{f}.$$

Let

$$\Delta^{*}:=\max_{i,j,m,n}|{\left({\hat{\Omega }}_{f}v_{m}\right)}^{\prime }\hat{\Gamma }\left({\hat{\Omega }}_{f}v_{n}\right)-\sigma_{ij}\Omega_{f}\left(m,n\right)|$$

denote the maximum discrepancy between the empirical and population covariance matrices. By the triangular inequality, we have

$$\begin{array}{cc} & \;|{\left({\hat{\Omega }}_{f}v_{m}\right)}^{\prime }\hat{\Gamma }\left({\hat{\Omega }}_{f}v_{n}\right)-\sigma_{ij}\Omega_{f}\left(m,n\right)|\\ & =|{\left({\hat{\Omega }}_{f}v_{m}\right)}^{\prime }\hat{\Gamma }\left({\hat{\Omega }}_{f}v_{n}\right)-{\left(\Omega_{f}v_{m}\right)}^{\prime }\Gamma \left(\Omega_{f}v_{n}\right)|\\ & \leq \underset{I}{\underset{︸}{|{\left({\hat{\Omega }}_{f}v_{m}\right)}^{\prime }\left(\hat{\Gamma }-\Gamma \right)\left({\hat{\Omega }}_{f}v_{n}\right)|}}+\underset{II}{\underset{︸}{|{\left({\hat{\Omega }}_{f}v_{m}-\Omega_{f}v_{m}\right)}^{\prime }\Gamma \left({\hat{\Omega }}_{f}v_{n}\right)|}}+\underset{III}{\underset{︸}{|{\left(\Omega_{f}v_{m}\right)}^{\prime }\Gamma \left({\hat{\Omega }}_{f}-\Omega_{f}\right)v_{n}|}}. \end{array}$$

Note that $∥{\hat{\Omega }}_{f}{∥}_{\infty }=O_{p}\left(1\right)$, by Lemma A9 (ii), we have

$$I\leq ∥{\hat{\Omega }}_{f}v_{m}{∥}_{\infty }^{2}{∥\hat{\Gamma }-\Gamma ∥}_{\infty }=O_{p}\left(\sqrt{\log p/T}\right).$$

By Lemma A2 (iii) and ${∥\Gamma ∥}_{\infty }=O\left(1\right)$, we have

$$II\leq ∥{\hat{\Omega }}_{f}v_{m}-\Omega_{f}v_{m}{∥}_{\infty }{∥\Gamma ∥}_{\infty }{∥{\hat{\Omega }}_{f}v_{n}∥}_{\infty }=O_{p}\left(\sqrt{\log T/T}\right).$$

Since $∥\Omega_{f}{∥}_{\infty }=O\left(1\right)$, we have

$$III\leq ∥{\left(\Omega_{f}v_{m}\right)}^{\prime }{∥}_{\infty }{∥\Gamma ∥}_{\infty }{∥\left({\hat{\Omega }}_{f}-\Omega_{f}\right)v_{n}∥}_{\infty }=O_{p}\left(\sqrt{\log T/T}\right).$$

The above results hold uniformly for i,j,m,n, thus we have

$$\Delta^{*}=O_{p}\left(\sqrt{\log p/T}+\sqrt{\log T/T}\right).$$

The rest of the proofs is similar to those in the proof of Theorem 2. We skip the details. ☐

**Proof of Theorem 5.** For a proof, see the proof of Theorem 3. ☐
